# Spatial variation of correct knowledge of the ovulatory cycle and its associated factors among reproductive age women in Ethiopia: geographically weighted regression analysis

**DOI:** 10.3389/frph.2025.1505749

**Published:** 2025-08-06

**Authors:** Werkneh Melkie Tilahun, Lamrot Yohannes Abay, Fantu Mamo Aragaw, Zenebe Abebe Gebreegziabher, Tigabu Kidie Tesfie

**Affiliations:** ^1^ Department of Public Health, College of Medicine and Health Sciences, Debre Markos University, Debre Markos, Ethiopia; ^2^ Department of Environmental and Occupational Health and Safety, College of Medicine and Health Sciences, Institute of Public Health, University of Gondar, Gondar, Ethiopia; ^3^ Department of Epidemiology and Biostatistics, Institute of Public Health, College of Medicine and Health Sciences, University of Gondar, Gondar, Ethiopia; ^4^ Department of Epidemiology and Biostatistics, School of Public Health, Debre Birhan University, Debre Birhan, Ethiopia

**Keywords:** knowledge, ovulatory cycle, women, spatial analysis, Ethiopia

## Abstract

**Background:**

Information about reproductive physiology like the ovulatory cycle helps women to understand their pregnancy risk and appropriately plan their pregnancies, which can reduce unintended pregnancy and the true risk of conception. Thus, this study aimed to explore the spatial variation of correct knowledge of the ovulatory cycle (KOC) across regions of Ethiopia and identify associated factors.

**Methods:**

A cross-sectional study design was employed based on the 2016 Ethiopian demographic and health survey. A total of 15,683 weighted samples were included. Geographically weighted regression and ordinary least square analysis were conducted. Models were compared using AICc & adjusted *R*^2^. A *P*-value of less than 0.05 was used to declare statistically significant spatial predictors.

**Results:**

In our study, only 23.58% [95% CI; 22.92–24.25%] of reproductive-age women had correct KOC. Significant hot spots were identified in Addis Ababa, Dire Dawa, and Hareri. Different factors showed a spatially significant effect on correct KOC. Media exposure showed positive effects ranging from 0.34 to 0.57 in the Somali, Amhara, Oromia, Addis Ababa, and SNNPR regions. Rich wealth status showed a positive spatial effect ranging from 0.13 to 0.54 in Benishangul Gumuz, most of Gambela, western Oromia, western Amhara, and Northwestern SNNPR. Proximity to health facilities had a positive effect ranging from 0.15 to 0.227 in Dire Dawa, Harari, eastern Oromia, and eastern and southeastern Somali. In most of the Amhara, Afar, Gambela, western and central Oromia, Benishangul, and Somali regions, education had a significant positive effect range of 0.23–0.36. In Dire Dawa, Harari, Somali, the majority of Oromia, and SNNPR regions, high community-level FP messages had a positive effect with a range of 0.28–0.39.

**Conclusion:**

In this study, the correct KOC among reproductive-aged women was found to be low. Significant spatial variation of the correct KOC among reproductive-age women was observed. Given the importance of formal education, rich household wealth status, media exposure, high community-level FP media exposure, and proximity to a health institution, area-based interventions that can take into account these important factors are needed to promote appropriate KOC.

## Introduction

Ovulation is a normal physiological process and phase of a woman's menstrual cycle among reproductive-aged women that occurs when an egg is developed and ready to be fertilized (1–3). The ovulation phase takes place on day 14 of the menstrual cycle due to the effect of estrogen on the production of luteinizing hormone (LH) by the pituitary gland, and having knowledge regarding the ovulation cycle is important to avoid unintended pregnancy or if they want to get pregnant (3, 4). Thus, accurate information about ovulation and other reproductive physiology, like the anatomy of the female body and the menstrual cycle, can reduce unintended pregnancy rates and the true risk of conception, which helps women understand their pregnancy risk and appropriately plan their pregnancies (1, 5).

Studies on women's reproductive health haven't focused much on knowledge of the ovulatory cycle (KOC) (2). Rather than focusing on the fertility window, many health educators instead emphasize the menstrual cycle and determining the menstrual phase (4). However, studies showed that knowledge regarding ovulation, fertility, and conception is limited among reproductive-age women. In the United States of America, 67.2% and 47.2% of reproductive-aged women did not know the ovulation timing or what ovulation is, respectively (1). Another study also declared that about 40% of reproductive-age women were unfamiliar with the ovulatory cycle (6). Likewise, a study from sub-Saharan Africa showed only 8.3% of reproductive-age women had correct KOC (4). Similarly, studies from Haiti (2) and Australia (7) declared that only 24.1% and 12.7% of women correctly identified their ovulation window.

Several previous studies revealed that age (4, 8–11), residence (2, 4, 9, 12), educational status (2, 4, 8–13), wealth index (2, 4, 8, 10, 12), occupation (2, 13), religion (10, 12, 13), marital status (9, 13), contraceptive use (2, 8, 9), knowledge of contraceptive methods (8), media exposure (13), having menstruation within the last 6 months (8, 9), pregnancy status (9), exposure to mass media family planning messages (2, 10, 12), fieldworker visit (2), community media exposure (8, 11), community family planning message exposure (10) were associated with correct KOC.

Different studies have been conducted in Ethiopia (8–10), but to the best of our knowledge, there are no previous studies in the literature addressing the geographical disparity of the correct KOC and spatial covariates that contributed to the geographical difference across regions of Ethiopia. Exploring the geographical disparity of the correct KOC and associated spatial covariates in the regions of Ethiopia is crucial to designing context- and area-based (local) interventions. For investigating spatial heterogeneity (when the spatial process being modeled has a different structure throughout the study area), Geographically Weighted Regression (GWR) analysis is an effective method (14). In order to model the remaining geographical autocorrelation and over-dispersion in the disease data that cannot be explained by the covariates, GWR is a useful method (15). Thus, the current study aimed to explain the spatial variation of correct KOC (areas with low and high prevalence of correct KOC) across regions of Ethiopia and identify associated factors using geographical weighted regression analysis.

## Method and materials

### Data sources, setting, and sampling design

The 2016 Ethiopian Demographic and Health Survey (EDHS) dataset was used for this cross-sectional study. The data was accessed from (http://www.dhsprogram.com) up on online request.

The survey was carried out in Ethiopia's nine regional states and two city administrations. It typically draws samples using a stratified, two-stage cluster sampling technique. The sampling frame includes 84,915 enumeration areas (EAs) from the 2007 Ethiopian Population and Housing Census (PHC). Each EA covers an average of 181 households. Following stratification of each region into urban and rural areas, a total of 645 EAs (202 in urban areas and 443 in rural areas) were selected with a probability proportional to EA size based on the 2007 PHC. A fixed number of 28 households from each cluster were chosen with equal probability through systematic selection. More details on data collection, samples, and survey questionnaires can be found in the 2016 EDHS report (16).

### Population

The source population consisted of all Ethiopian women between the ages of 15 and 49. The study population consisted of all women of reproductive age who spent the night before the survey and lived in the selected enumeration areas (EAs) and households. Our study was based on datasets from women (individual record files). The study included a weighted sample of 15,683 reproductive-age women. However, reproductive-aged women from clusters without coordinate data (“0” coordinate data) were excluded from spatial analysis.

### Variables of the study

#### Outcome variable

The outcome variable was correct knowledge of the ovulation cycle (KOC) among reproductive women (“yes” or “no”). Women were asked, “When is the ovulation time?” Response options were designated as “during her period,” “after her period ended,” “middle of the cycle,” “before the period begins,” “at any time,” and “don't know”. The outcome variable was recoded, and respondents who indicated “middle of the cycle” were considered to have the correct KOC and coded as “1,” and otherwise respondents were considered to have the incorrect KOC and coded as “0” (9, 17).

#### Independent variables

A variety of literature reviews were conducted in order to select independent variables. The following individual-level characteristics were included: age, wealth index, education level, use of contraceptives, awareness of contraceptive techniques, having a period during the previous 6 months, exposure to family planning messages, and fieldworker visits. Additionally, community-level characteristics including community-level media exposure to local media and FP messages were incorporated.

### Measurement and operational definitions

Household media exposure was created by combining whether women read a newspaper or magazine, listened to the radio, or watched television and coded as “yes” (if a woman has been exposed to at least one of these media) or “no” (if she has not).

Exposure to mass media family planning messages was created by combining four variables: “have heard about family planning messages on radio in the last few months”, “have heard about family planning messages on television in the last few months”, “have heard about family planning in a newspaper or magazine in the last few months”, “have heard about family planning by text messages on a mobile phone in the last few months”, and “has the fieldworker visited in the last 12 months and talked about family planning”. These five variables were combined and recoded as “yes” if a woman heard family planning messages through at least one of these mass media, and “no” if she did not hear family planning messages through any of the mass media (2).

Community-level media exposure: an aggregated variable from household media exposure measured as the proportion of women who had been exposed to at least one media (newspaper or magazine, radio, or television) and categorized based on the median value as low (communities with <50% of women exposed) and high (communities with ≥50% of women exposed).

Community-level FP message exposure: aggregated variable from exposure to mass media FP messages and measured as the proportion of women who had been exposed to FP messages at least through one mechanism: newspaper or magazine, radio, television, text messages on mobile phones, and fieldworker messages, and categorized based on the median value as low (communities with <50% of women exposed) and high otherwise.

### Data management and analysis

Data extraction, re-coding, visualization, and other statistical analyses were carried out using Excel, Stata version 16, and Arc-GIS version 10.8 after getting the data from the MEASURE DHS website. Descriptive statistics were used and were reported in the form of frequency, percentage, text, figures, and tables. Sampling weight (v005/1,000,000) was an adjustment factor used in tabulations to account for differences in the likelihood of selection and interview between cases in a sample owing to design or chance.

#### Descriptive spatial analysis

Detecting geographical clustering in data sets is critical in spatial data analysis (18). Global Moran's *I* is a widely used global index that assesses the similarity of values in nearby locations to an overall mean value and reflects a spatially weighted version of Pearson's correlation coefficient (19). The value ranges between −1 and 1, with values around −1 suggesting that the event was scattered, but values near +1 indicate that the event was clustered and distributed randomly if Moran's *I* value is zero. The presence of spatial autocorrelation is confirmed by a statistically significant Moran's *I* (*P* < 0.05) (20). Spatial autocorrelation analysis was performed to determine whether the spatial pattern of correct KOC among reproductive-age women in Ethiopia was clustered, dispersed, or random.

#### Hot-spot analysis and empirical Bayesian kriging interpolation mapping

Local indicators of spatial autocorrelation, like the Gettis-Ord Gi* statistic, can provide more information by estimating the distribution of events at the local level (21). The Gettis-Ord Gi* statistic calculates a *Z*-score and *P*-value for each grid cell and identifies statistically significant hot spots. Finally, the interpolation mapping technique with empirical Bayesian Kriging kernel density estimation was applied for smoothing the hotspot analysis. Hence, the use of Bayesian approaches for the identification of hotspots is increasing due to their capacity to reduce false positives and false negatives by 50% compared to classical methods (22).

#### Spatial scan statistics

A Bernoulli-based model spatial scan analysis using Kuldorff's SaTScan version 9.6 software was conducted to identify significant primary and secondary clusters of correct knowledge of the ovulatory cycle. A circular scanning window that goes across the region of Ethiopia means that women who correctly respond to the period of the ovulatory cycle were considered cases, while those who didn't respond correctly were considered controls. A maximum spatial cluster size of <50% of the population was used as an upper limit. The most likely clusters were identified using likelihood ratio tests and their corresponding significance levels based on the default 999 Monte Carlo replications.

#### Spatial regression analysis

Spatial regression models are critical for determining the relationship between the density of particular events and other environmental, demographic, and socioeconomic factors in the population (23). As a result, we sought to comprehend the relationship between the prevalence of correct KOC determined at each cluster and the other nine (9) explanatory variables from previous studies and preliminary multilevel analysis chosen based on expert opinion, as well as their importance during the multilevel analysis. We began our spatial regression modeling with OLS regression, which requires residuals to be normally distributed independently and identically. Furthermore, the residuals are presumed to be homoscedastic. The OLS regression model has numerous model diagnostics, including *R*^2^, adjusted *R*^2^, VIF, the Jarque–Bera statistic, the joint *F* statistic, the joint Wald statistic, and the Koenker (BP) statistic.

The GWR model was conducted with similar dependent and explanatory variables as the global model. The GWR has a geographical weighting system for the features included in the local regression equations. Near features and features that are farther away from the regression point have more weight and less weight in the regression equation, respectively. These weights are determined by a distance decay function called the kernel (24). In ArcGIS, there are two types of kernels: fixed and adaptive. The spatial configuration of the feature is the main reason to choose the kernel type. For reasonably or regularly positioned observations, a fixed kernel is appropriate. However, if observations are clustered, an adaptive kernel is appropriate (14).

Another important parameter to be considered in GWR is bandwidth (neighborhood). It is the distance band or number of neighbors used for each local regression equation that controls the degree of smoothing in the model (24). In ArcGIS, there are three choices for the corrected Akaike information criteria (AICc), cross-validation (CV), and bandwidth parameter. The AICc method automatically finds a bandwidth with the minimum AICc, while the CV method finds a bandwidth that minimizes the CV score. In practice, there isn't much to choose between the two methods, although the AICc is our preferred method. The AICc method can reduce model complexity due to the number of variables included in the study and the bandwidth (14).

In our study, observations showed significant spatial clustering, and the explanatory variables included in the model were nine. Thus, in order to reduce model complexity, we were interested in using the adaptive kernel type determined by AICc. Comparison between OLS and the GWR model was made using adjusted *R*^2^ and AICc values. A model with a high value of adjusted *R*^2^ and a low AICc value was the preferred model (i.e., GWR). If we get a less than 4 AICc difference between two models, we cannot choose one of them, but if this difference is greater than 10, there is evidence to choose a model with a small AICc value (25). Finally, a spatial autocorrelation test was conducted among the residuals of the GWR model to determine whether the residuals are randomly distributed after we controlled the spatial dependencies present in the residuals of the OLS model.

### Missing values

Any missing data in the dataset was managed according to DHS guidelines. Thus, the final model was based on complete observation.

### Ethical considerations

The study was a secondary data analysis based on the publicly available DHS datasets; thus, ethical approval and participant consent were not necessary. However, we requested the data from the MEASURE DHS Program, and permission was granted to download and use the data.

## Results

A total of 15,683 weighted samples were included. About 21.56% of the respondents were in the age bracket of 15–19 years. Nearly half of the participants (47.81%) had not attended formal education. The majority (66.72%, 65.19%, 68.66%, and 74.66%) of the participants were unemployed during data collection, in a union, not exposed to FP messages, and not using contraceptives, respectively. Almost all (98.32 and 92.76) are knowledgeable about contraceptive methods and not currently pregnant. About 36.35%, 75.20%, 55.57%, 77.84%, and 57.46% were from the Oromia region, had not been visited by fieldworkers in the last 12 months, experienced menstruation in the last 6 weeks, were residing in rural areas, and were from clusters with low community FP message exposure, respectively (Table 1).

**Table 1:** Characteristics of the respondents, EDHS 2016.

Variables	Categories	Weighted frequency (15,683)	Percentage (%)
Maternal age	15–19	3,381	21.56
	20–24	2,762	17.61
	25–29	2,957	18.85
	30–34	2,345	14.95
	35–39	1,932	12.32
	40–44	1,290	8.22
	45–49	1,017	6.48
Education level	No education	7,498	47.81
	Primary	5,490	35.01
	Secondary	1,818	11.59
	Higher	877	5.59
Religion	Orthodox	6,786	43.27
	Protestant	3,674	23.43
	Muslim	4,893	31.20
	Other	330	2.10
Household media exposure	No	8,793	56.07
	Yes	6,890	43.93
Wealth index	Poorest	2,633	16.79
	Poorer	2,809	17.91
	Middle	2,978	18.99
	Richer	3,100	19.76
	Richest	4,163	26.55
Currently employed	No	10,463	66.72
	Yes	5,220	33.28
Marital status	Never in union	4,036	25.74
	Currently in union	10,223	65.19
	Formerly in union	1,423	9.08
Exposure to FP messages (medias)	No	10,768	68.66
	Yes	4,915	31.34
Current contraceptive use	No	11,709	74.66
	Yes	3,974	25.34
Knowledge of contraceptive methods	No	264	1.68
	Yes	15,420	98.32
Currently pregnant	No	14,548	92.76
	Yes	1,135	7.24
Visited by fieldworker in last 12 months	No	11,794	75.20
	Yes	3,890	24.80
Menstruated in last 6 weeks	No	6,969	44.43
	Yes	8,714	55.57
Residence	Urban	3,476	22.16
	Rural	12,207	77.84
Region	Tigray	1,129	7.20
	Afar	128	0.82
	Amhara	3,714	23.68
	Oromia	5,701	36.35
	Somali	459	2.93
	Benishangul	160	1.02
	SNNPR	3,288	20.97
	Gambela	44	0.28
	Harari	39	0.25
	Addis Ababa	930	5.93
	Dire Dawa	90	0.58
Distance to health facility	Big problem	7,890	50.31
	Not big problem	7,793	49.69
Community level media exposure	Low	8,136	51.88
	High	7,547	48.12
Community level poverty	Low	8,373	53.39
	High	7,310	46.61
community FP message exposure	Low	9,012	57.46
	High	6,671	42.54

### Magnitude of correct KOC

**Figure 1:**
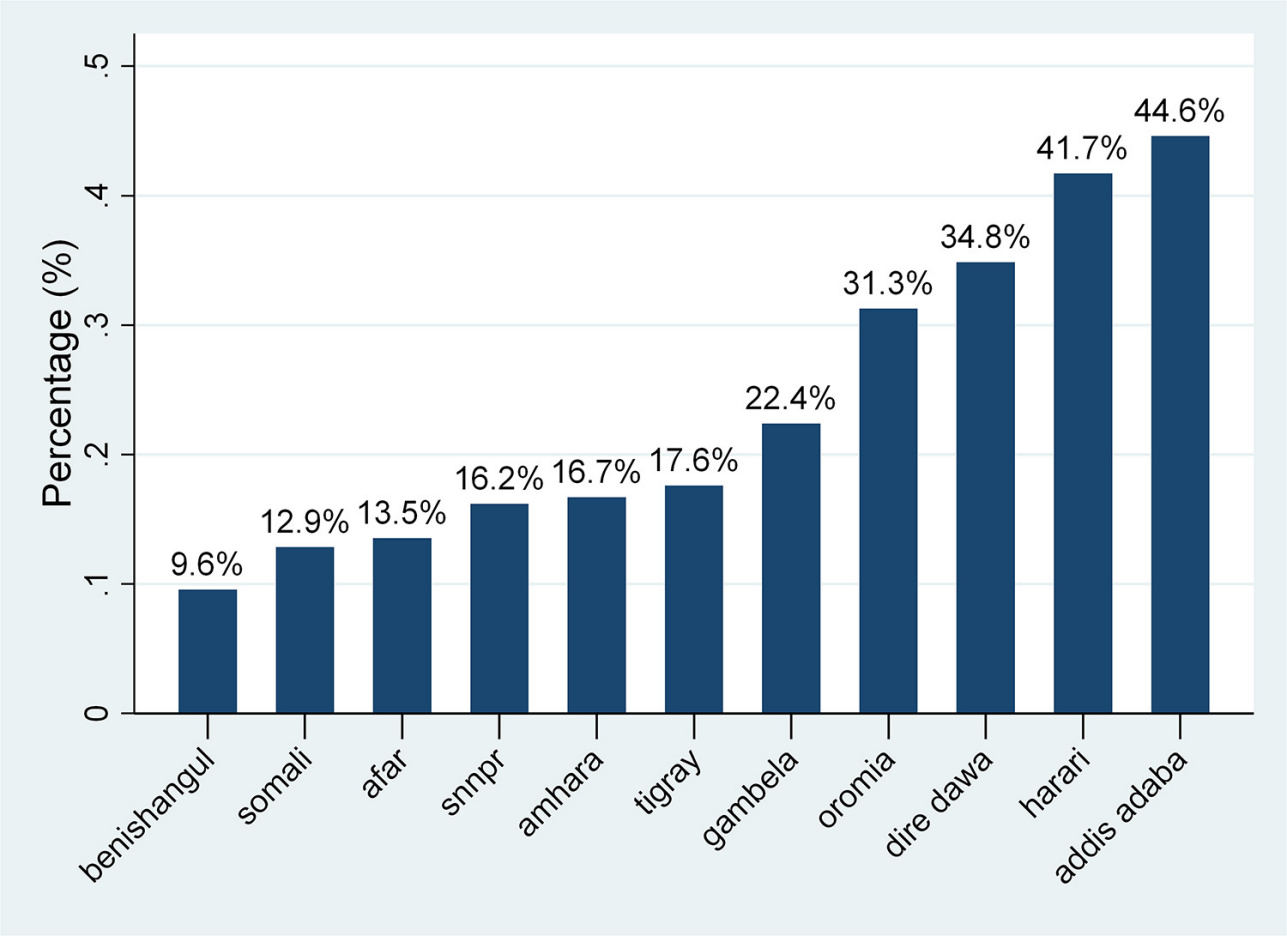
Prevalence of correct KOC among reproductive-aged women across regions of Ethiopia, 2016.

The overall prevalence of correct KOC among reproductive-age women was found to be 23.58% [95% CI: 22.92–24.25%], with the highest prevalence in Addis Ababa (44.6%) and lowest in the Benishangul region (9.6%) (Figure 1).

### Spatial distribution and hot spot analysis of correct KOC

**Figure 2:**
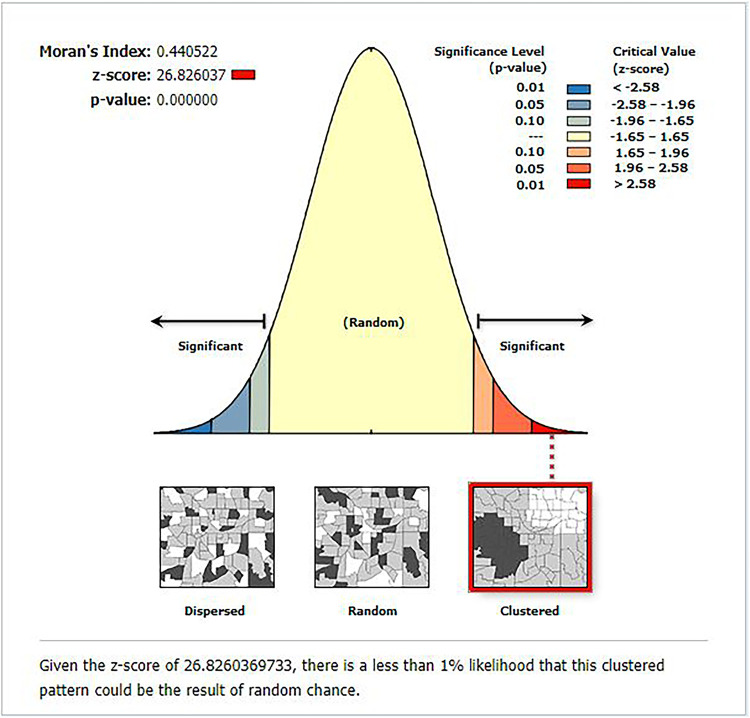
Global spatial autocorrelation analysis of correct KOC among reproductive age women in Ethiopia, 2016.

**Figure 3:**
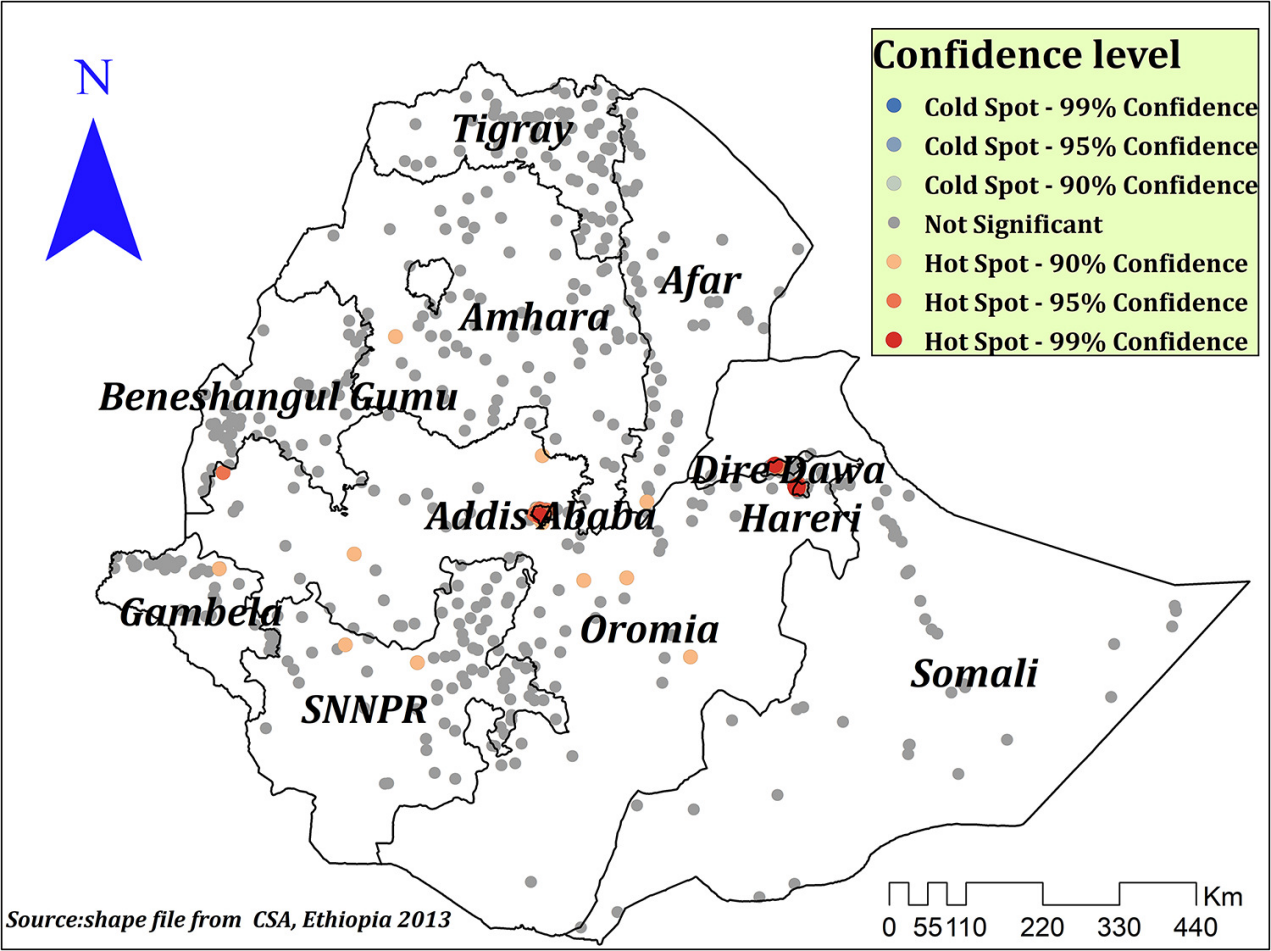
Hot spot analysis of correct KOC among reproductive age women in Ethiopia, 2016.

The spatial autocorrelation analysis indicated the presence of significant spatial clustering of the correct KOC across the country with a significant global Moran's index (*I*) of 0.441 (*P*-value = 0.000) and *Z*-score of 26.83 (Figure 2). During hot spot analysis, the Getis-Ord Gi* showed significant hot spot areas in Addis Ababa, Dire Dawa, and Hareri (Figure 3). This indicates that there was a clustering of high prevalence of correct KOC among reproductive-age women in the mentioned areas.

### Spatial prediction of correct KOC among reproductive age women in Ethiopia

**Figure 4:**
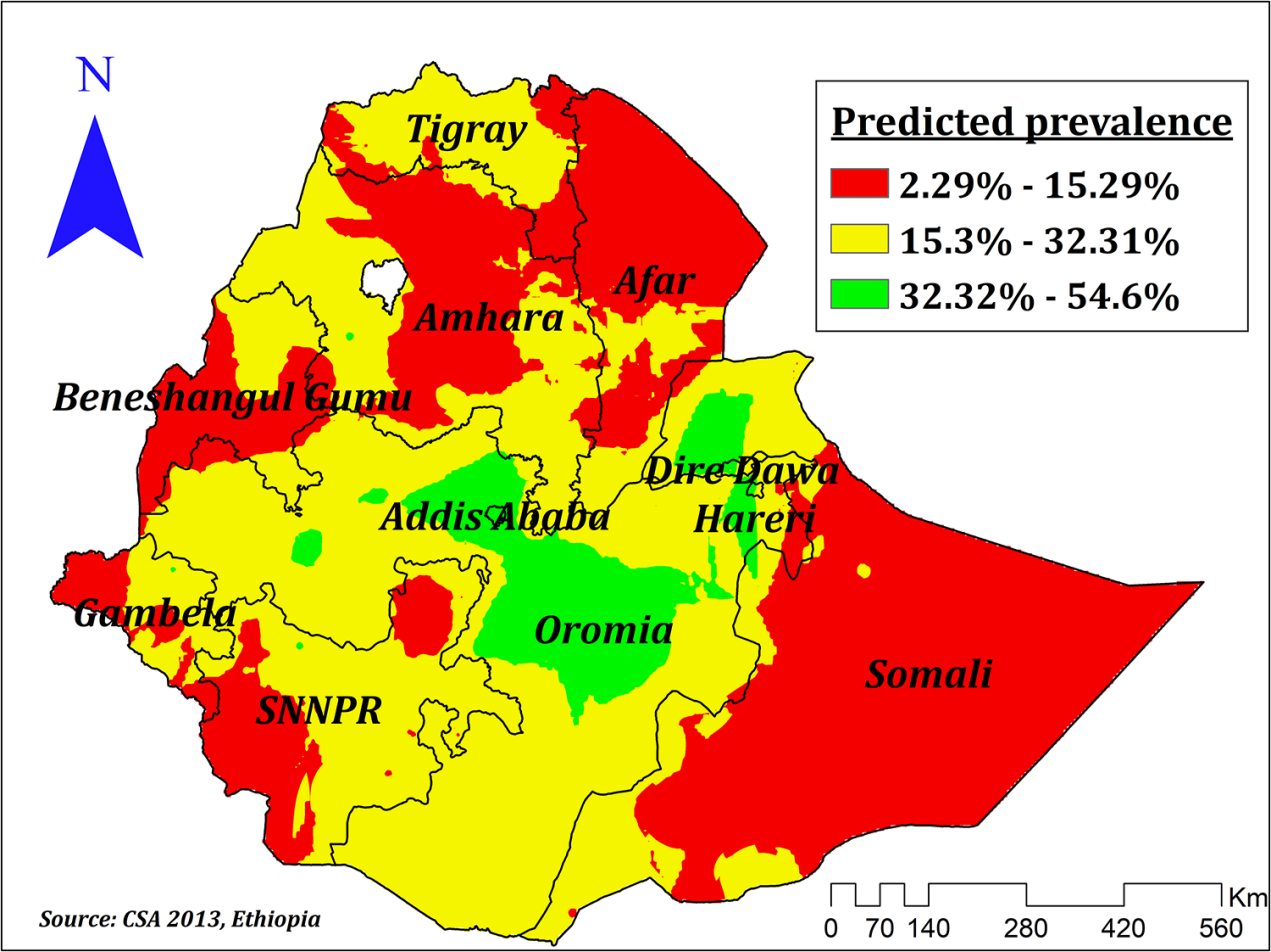
Empirical Bayesian kriging interpolation of prevalence of correct KOC among reproductive age women in Ethiopia.

The empirical Bayesian Kriging interpolation predicted low and high prevalence areas of correct KOC among reproductive-age women. Accordingly, the majority of Benishangul, Somali, Afar, Amhara, parts of SNNPR, some parts of Tigray, and Gambela regions were predicted to have a low prevalence of correct KOC, while Addis Ababa, some parts of Oromia, Dire Dawa, and Hareri were predicted to have a high predicted prevalence of correct KOC (Figure 4).

### Spatial scan analysis

**Figure 5:**
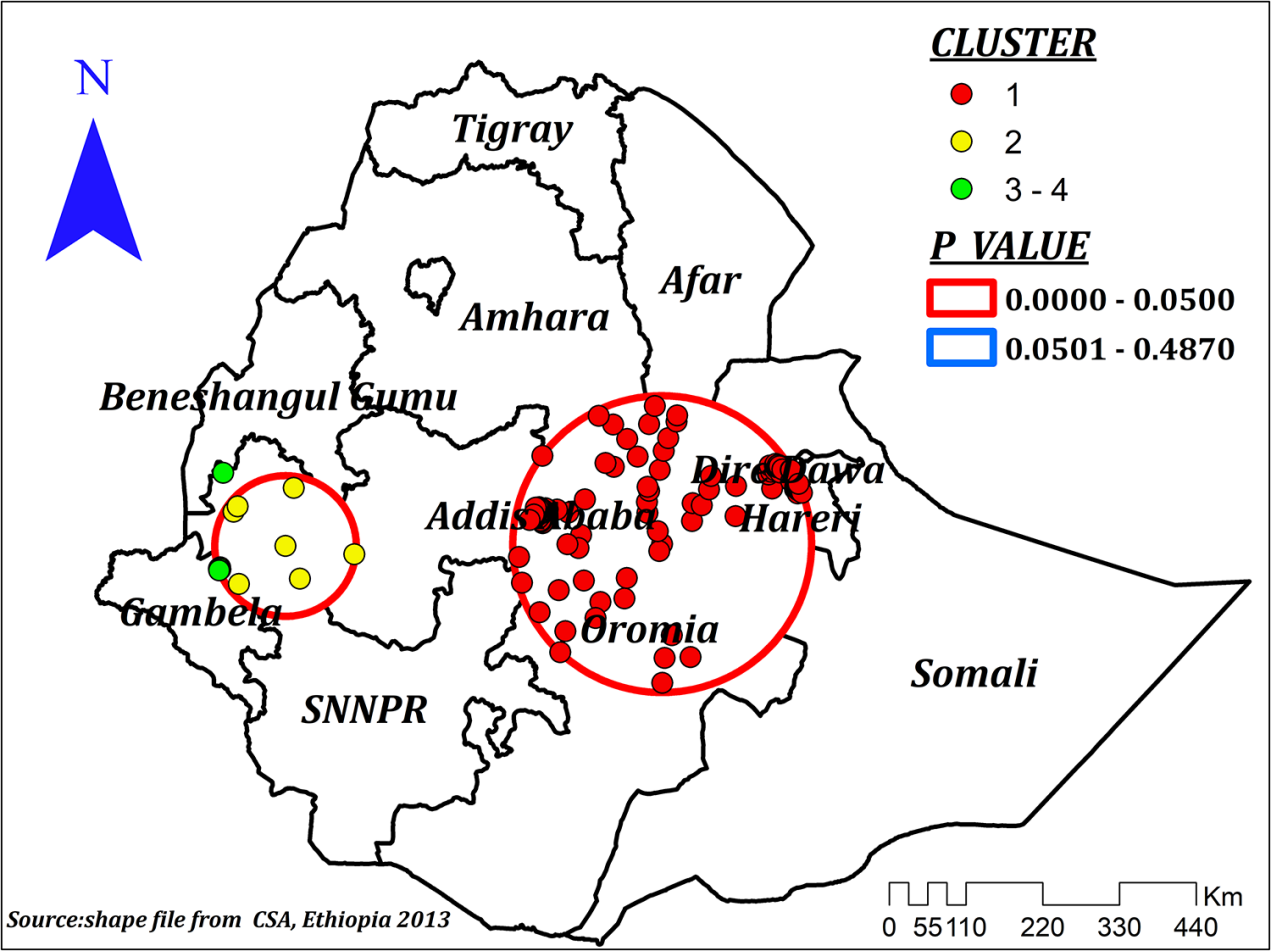
Spatial scan analysis showing most likely (primary) and secondary cluster for correct KOC among reproductive age women, Ethiopia.

Spatial scan statistics identified a total of 199 significant clusters. Of those, 184 were most likely (primary), and the rest, 15, were secondary clusters. The primary clusters were located at 8.591911 N, 40.352665 E/213.80 km radius in southern Amhara and Afar, Addis Ababa, Dire Dawa, Harari, central, and eastern Oromia, and women residing in these clusters were 2.4 times more likely to have the correct KOC compared to those women residing outside the window (Figure 5). The second significant clusters were located at 8.553292 N, 35.448128 E/101.17 km in western Oromia and eastern Gambela and those women residing in these clusters were 45% more likely to have correct KOC than women outside the window (Table 2).

**Table 2:** Spatial scan analysis of correct KOC in Ethiopia using 2016 EDHS data.

	Cluster type	
	Primary cluster	Secondary cluster
Significant clusters detected	245, 529, 122, 71, 476, 49, 319, 506, 51, 412, 230, 123, 333, 564, 372, 125, 149, 491, 213, 524, 121, 484, 624, 452, 39, 93, 290, 40, 201, 562, 336, 303, 295, 438, 472, 286, 90, 402, 353, 102, 83, 211, 287, 330, 560, 236, 509, 428, 453, 155, 252, 135, 19, 539, 451, 264, 639, 247, 61, 293, 15, 464, 475, 302, 261, 153, 225, 582, 441, 414, 159, 170, 110, 557, 305, 635, 144, 112, 195, 108, 59, 532, 369, 626, 91, 463, 31, 637, 100, 107, 314, 645, 37, 11, 339, 487, 619, 608, 594, 274, 145, 25, 147, 30, 43, 380, 644, 282, 546, 74, 166, 111, 151, 273, 190, 631, 535, 519, 283, 101, 467, 471, 363, 140, 613, 5, 289, 606, 390, 185, 444, 522, 385, 202, 224, 27, 514, 493, 352, 311, 473, 217, 310, 607, 115, 513, 173, 54, 443, 614, 179, 133, 393, 28, 29, 228, 580, 500, 60, 56, 397, 383, 418, 58, 257, 240, 157, 610, 44, 534, 387, 238, 396, 587, 329, 483, 194, 321, 523, 26, 454, 423, 242, 495	558, 555, 304, 448, 248, 175, 63, 114, 411, 47, 469, 291, 549, 221, 231
Coordinates/Radius	8.591911 N, 40.352665 E/213.80 km	8.553292 N, 35.448128 E/101.17 km
Population	4,829	384
Number of cases	1,915	131
Relative risk	2.40	1.45
Percent cases in area	39.7	34.1
Log likelihood ratio	462.15	10.52
*P*-value	<0.00000000000000001	0.018

#### The ordinary least square regression analysis results

The OLS model showed a significant joint *F* and Wald statistic, indicating that the overall model is significant. The Jarque–Bera statistic was significant (*P* = 0.001), indicating the predicted values of the model were biased and residuals were not randomly distributed across areas. The Koenker statistic reported as the Breusch–Pagan heteroscedasticity coefficient was significant (*P* = 0.000). Which indicates the presence of an inconsistent relationship between the prevalence of correct KOC and other explanatory variables due to non-stationarity or heteroscedasticity (Table 3).

**Table 3:** OLS regression analysis results, EDHS 2016.

Variable	Coefficient	Robust Std. error	Robust probability	VIF
Intercept	0.037	0.034	0.282	-
Proportion of women with age greater than or equal to 30 years	0.004	0.0048	0.930	1.11
Proportion of women with primary and above education	0.060	0.034	0.081	3.27
Proportion of women from household with richer and above wealth quintile	0.104	0.027	**0.000**	4.62
Proportion of women from household with media exposure	0.198	0.031	**0**.**000**	4.53
Proportion of women without a big problem of distance to health facility	−0.043	0.022	0.052	1.79
Proportion of women who were using contraceptive	−0.085	0.04	**0**.**034**	1.39
Proportion of women with no knowledge of any type of contraceptive	−0.048	0.037	0.195	1.40
Proportion of women who had menstruated in the last 6 weeks	0.088	0.045	**0**.**049**	1.77
Proportion of women from cluster of high community FP messages.	0.020	0.015	0.181	2.00
OLS diagnostics				
Diagnostic parameters	Value		*P*-value	
Number of observations	622		-	
Joint F statistic	57.25		**0.000**	
Joint Wald statistic	519.58		**0**.**000**	
Koenker (BP) statistic	53.68		**0**.**000**	
Jarque Bera statistic	14.35		**0**.**001**	
Model comparison				
Model parameter	OLS (global) model		GWR	
*R*^2^ (%)	45.71		59.85	
Adjusted *R*^2^ (%)	44.91		54.92	
AICc	−703.62		−794.48	

Bold indicates *P*-values <0.05.

Our OLS output indicated to us to ensure whether there is spatial autocorrelation in the residuals. Accordingly, residuals from the OLS model were spatially and significantly autocorrelated, which violates one of the assumptions of OLS regression analysis and indicates the modeling process is spatially heterogeneous or non-stationary. Thus, the results of the OLS regression are unreliable, and it is necessary to make reliable predictions. In order to account for the heterogeneous relationship of the process and improve the reliability of the predictions of the effect size of explanatory variables, we need to have GWR to account for the spatial autocorrelation and varying relationships across space.

The Variance Inflation Factor (VIF) measures collinearity or redundancy among explanatory variables considered in the model. A rule of thumb regarding the cutoff value for VIF is 7.5. Thus, an explanatory variable with a VIF value greater than 7.5 should be removed (26). However, in our OLS model, all explanatory variables had a VIF value of less than 7.5; thus, multicollinearity was not a serious problem in further analysis.

#### Geographical weighted regression

**Figure 6:**
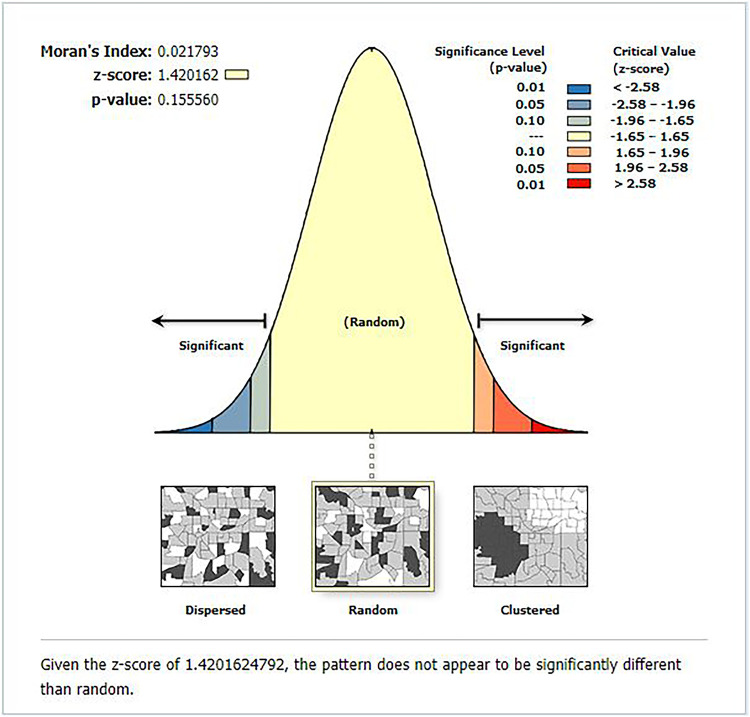
The global spatial autocorrelation analysis of residuals after geographical weighted regression analysis.

Explanatory variables included in the global model were used for the GWR analysis. Compared to the global model, the GWR model was better. The adjusted *R*^2^ in the GWR increased from 44.9 in the OLS to 54.9. This means that the GWR model explained an additional 10% of the variation in the prevalence of correct KOC across regions. Moreover, the AICc value for the OLS model was −703.62, and it decreased to a value of −794.48 in the GWR (difference = 90.86). Thus, we can conclude that the later model well explained the spatial heterogeneity. We checked the spatial autocorrelation of the residuals before interpreting the GWR result. The autocorrelation of the residuals was randomly distributed; hence, Moran's *I* for the residuals was 0.022 (*P* = 0.156). As Figure 6 shows little evidence of autocorrelation, which indicates that the explanatory variables considered in our model well explained the spatial dependencies that have been present in the residuals for the global model. Therefore, our model was well specified.

**Figure 7:**
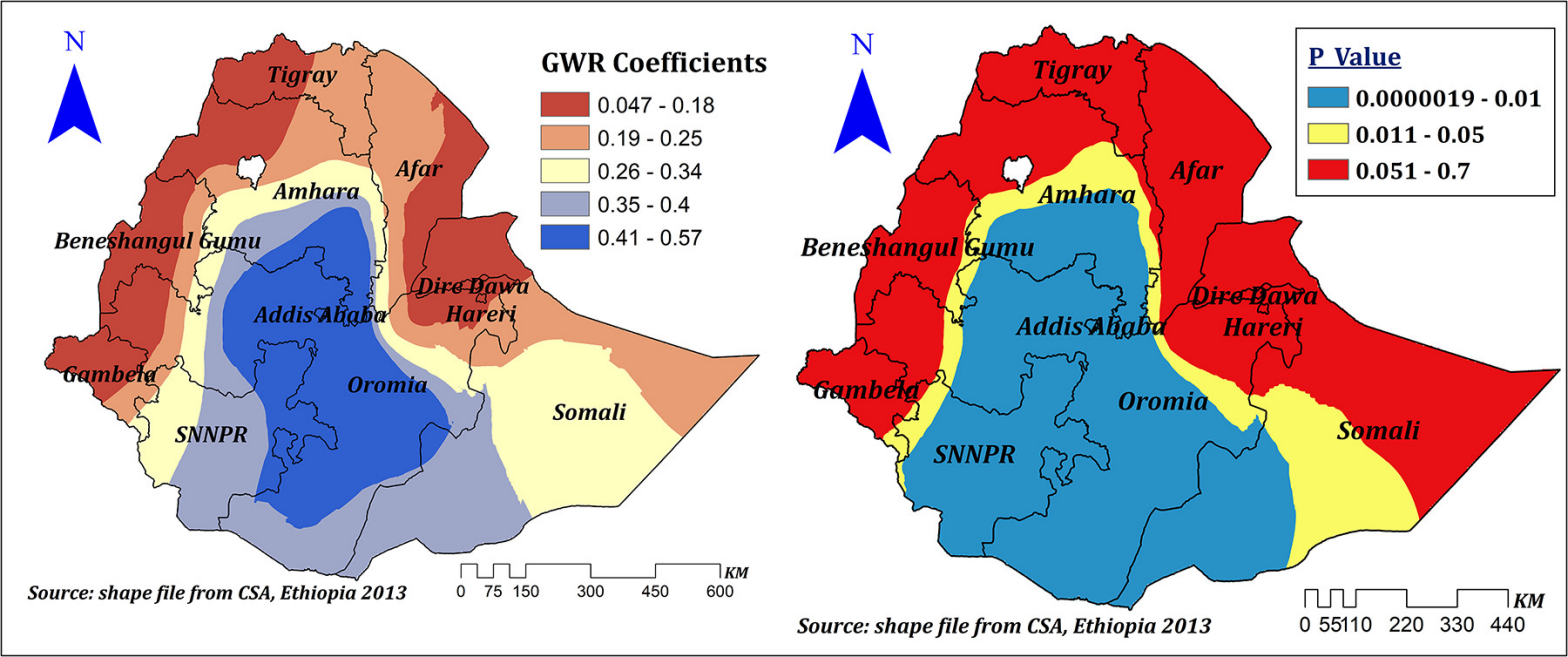
The GWR coefficients of household media exposure predicting correct KOC among reproductive age women in Ethiopia, 2016.

Household media exposure had a positive effect (ranges between 0.047 and 0.57) on the prevalence of correct KOC among reproductive-age women in Ethiopia. However, its statistical significance varies across different regions of Ethiopia. In the significant areas of Amhara, Oromia, Addis Ababa, SNNPR, and Somali regions, a 1% increase in the proportion of women with media exposure increases the prevalence of correct KOC by a range of 34%–57%. However, household media exposure had no significant effect in Dire Dawa, Hareri, almost the entire Tigray and Afar, as well as the majority of the regions of Benishangul, Gambela, and Somali (Figure 7).

**Figure 8:**
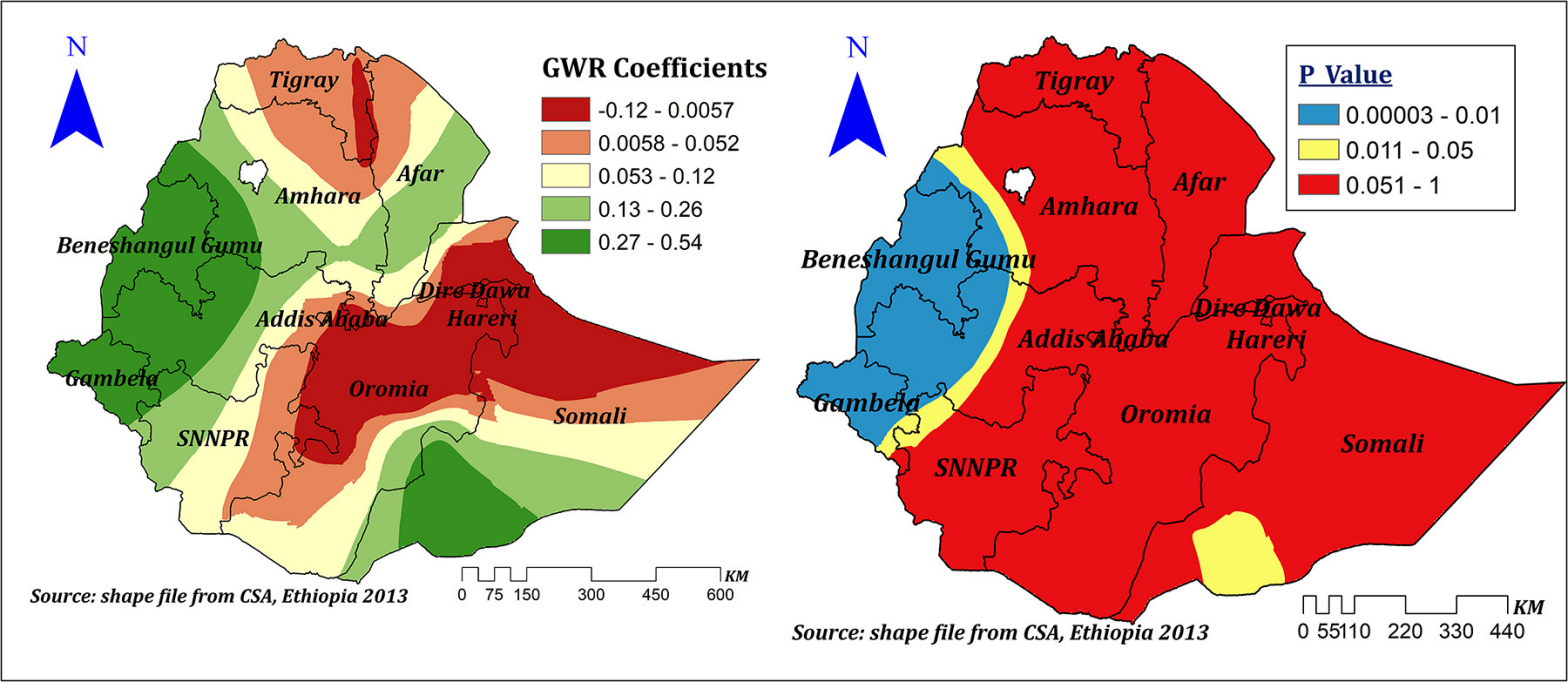
The GWR coefficients of rich household wealth quintile predicting correct KOC among reproductive age women in Ethiopia, 2016.

Being from a household with a rich wealth status indicated a positive and negative spatial effect on correct KOC among reproductive-age women in Ethiopia, which ranges from −0.12 to 0.54. However, its significant effect was observed in the entire Benishangul Gumuz, the majority of Gambela and western Oromia, and some parts of western Amhara and the Northwest SNNPR. In significant areas, a 1% increase in the proportion of women from households with a rich wealth quintile increases the prevalence of correct KOC among reproductive-age women by a range of 13%–54% (Figure 8).

**Figure 9:**
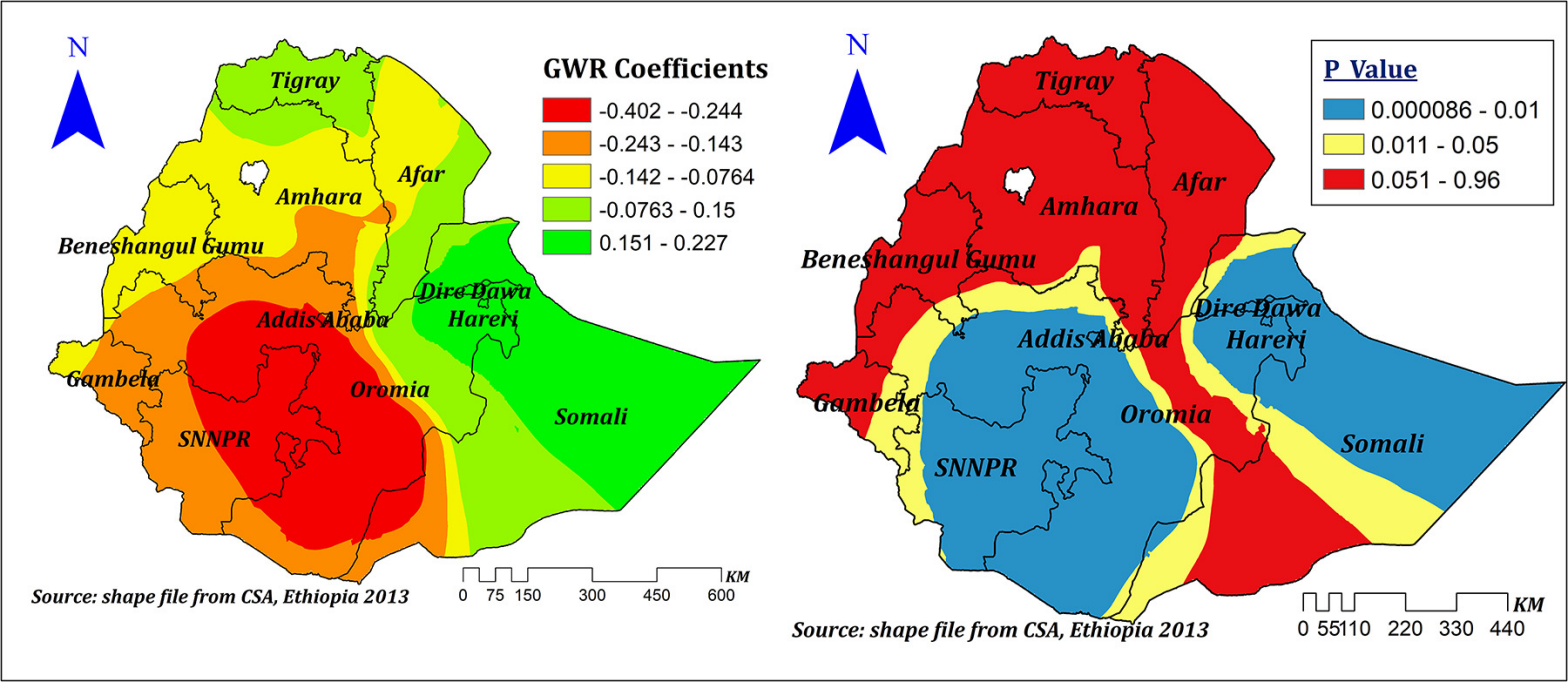
The GWR coefficients and spatially varying values of significance level of no problem of distance to health facility predicting correct KOC in the final model.

Distance to health facilities showed a positive and negative spatial effect on correct KOC among reproductive-age women in Ethiopia, which ranges between −0.402 and 0.227 with different corresponding significance levels. It showed a significant negative effect in Addis Ababa, the majority of SNNPR, Oromia, some parts of Gambela, Benishangul Gumuz, Amhara, and Southwest Somali, and ranges between −0.243 and −0.402, which indicates a 1% increase in the proportion of women residing near to health facilities corresponded to a 24.3%–40.2% decrease in the prevalence of correct KOC. On the other hand, in some other significant areas, such as Dire Dawa, Harari, eastern Oromia, and eastern and southeastern Somali, it showed a significant positive effect that ranged between 0.15 and 0.227. Thus, a 1% increase in the proportion of women residing near health facilities corresponded to a 15%–22.7% increase in the prevalence of correct KOC in these areas; other variables in the model remain constant (Figure 9).

**Figure 10:**
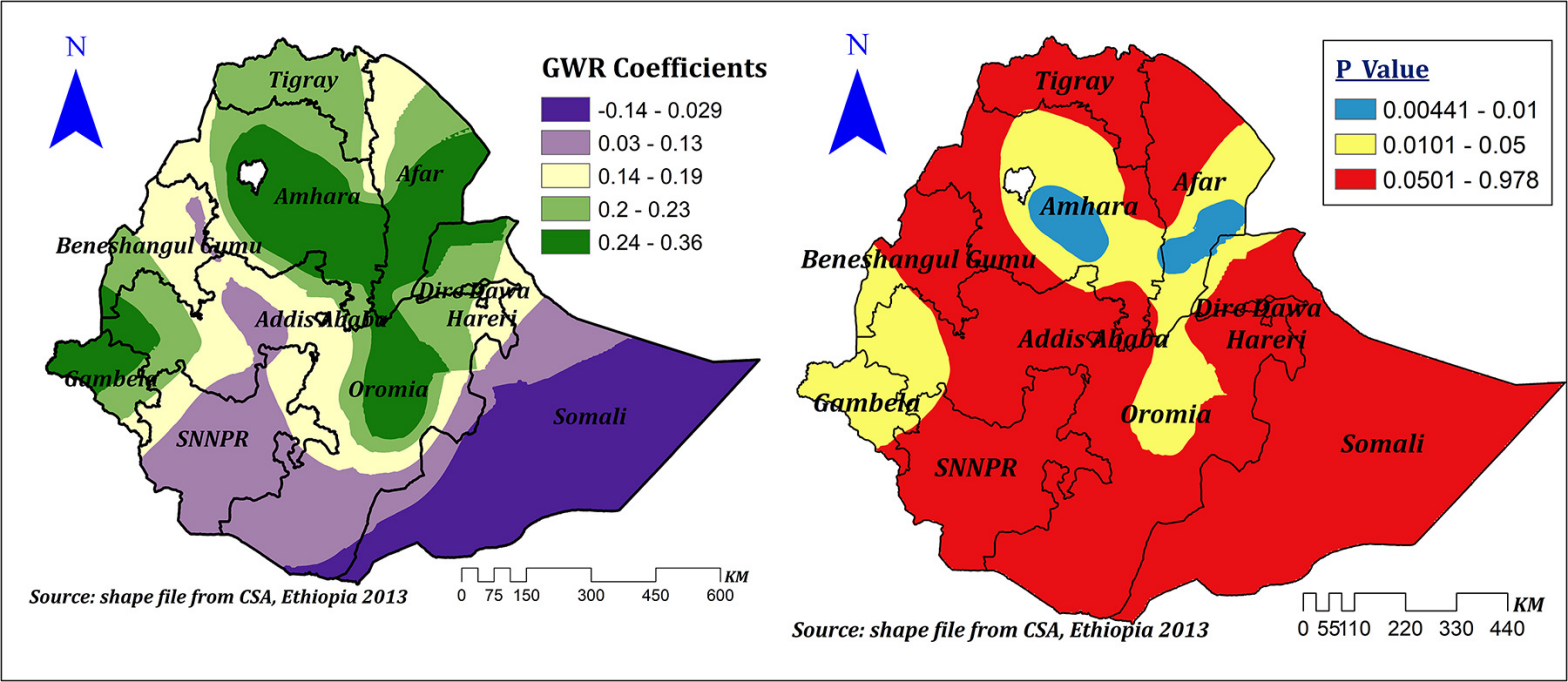
The GWR coefficients and spatially varying significance level of education for predicting correct KOC among reproductive age women in Ethiopia.

The effect size of education was statistically significant, varied across regions of Ethiopia, and ranged between −0.14 and 0.36, indicating that education had a negative and positive spatial effect on the prevalence of correct KOC. However, the significant effect was only seen in the majority of the Amhara, Afar, and Gambela regions and in some parts of western and central Oromia, Benishangul, and Somali regions. In these significant regions, a 1% increase in the proportion of women with primary and above education increases the prevalence of correct KOC by a range of 23%–36%, keeping other variables constant (Figure 10).

**Figure 11:**
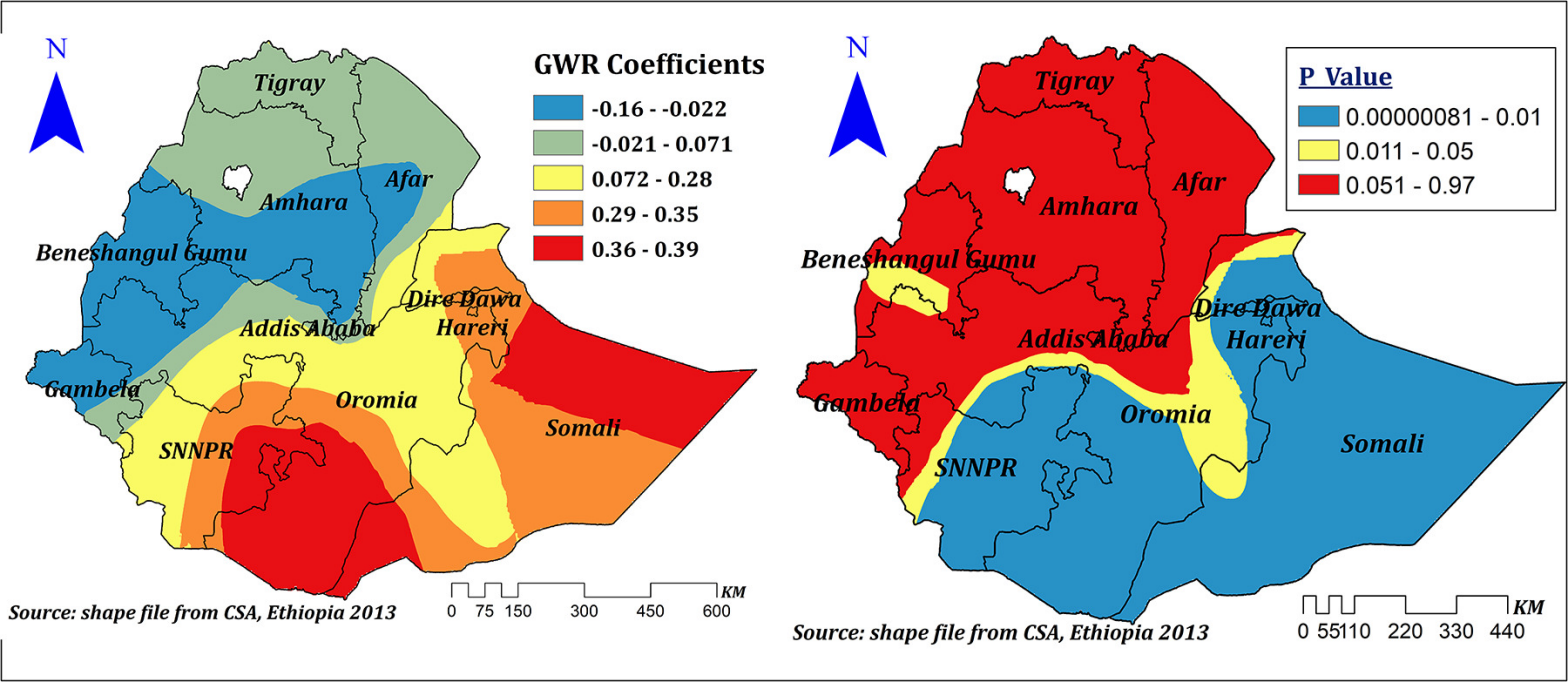
The GWR coefficients and spatially varying significance level of high community level FP messages predicting correct KOC among reproductive age women in Ethiopia.

High community-level FP messages had a spatially positive and negative effect on correct KOC ranges between −0.16 and 0.39. However, in the significant areas (Dire Dawa, Harari, Somali, majority of Oromia, and SNNPR), the effect size ranges between 0.28 and 0.39. This means a 1% increase in the proportion of women in clusters of high community FP messages in these areas can increase the correct KOC by a range of 28%–39%. Surprisingly, in parts of Benishangul and western Oromia, when the proportion of women from clusters of high community FP messages increases by 1%, correct knowledge of KOC decreases by a range of 2.2%–16% (Figure 11).

## Discussion

This study examined the geographical variation of correct KOC and its associated factors among reproductive-aged women in Ethiopia to understand the regional variation and distribution as well as factors contributing to the observed regional variation. In spatial regression analysis, having formal education, rich household wealth status, household media exposure, high community-level family planning (FP) media exposure, and proximity to a health facility were significant explanatory variables for the spatial variation of the correct KOC.

In our study, only 23.58% [95% CI: 22.92–24.25%] of reproductive-age women had the correct KOC. Similar findings were reported from Haiti (2) and Africa (5). However, our finding is higher than previous findings from Africa (11), sub-Saharan Africa (4), and Australia (7, 27). This might be due to a difference in the sociocultural context (4). Our findings are also lower than those of a study conducted in the United States (1, 6). This might be due to poor communication and information-seeking behavior about sexual matters in the family, school, and community among women in Africa (4, 28, 29).

Our study also showed that the spatial distribution of the correct KOC was non-random and clustered spatially at the cluster level. High clustering was observed at Addis Ababa, some parts of central, eastern, and western Oromia, Harari, Dire Dawa, and some parts of the northwest Somali region. Similarly, a high proportion of clustering was observed among the 199 clusters during the spatial scan analysis, with significant clustering in southern Amhara and Afar, Addis Ababa, Dire Dawa, Harari, central as well as western Oromia, and some parts of the Northwest Somali region. However, during interpolation, the majority of the Somali, Afar, SNNPR, Benishangul, and Amhara regions, as well as some parts of the Gambela and Tigray regions, were predicted to have the lowest prevalence of correct KOC. Previous studies conducted in Africa also declared the geographical variation of KOC (4, 30). This might be due to socioeconomic, demographic, cultural, behavioral, and lifestyle differences among participants across regions (31–33). Disparities in the implementation of different health-related programs across regions may also contribute to variations in women's knowledge of their ovulation period and health care-seeking behavior as a whole (34). Inequities between and within regions of Ethiopia in the use of maternal health services may also contribute to the observed variation (35). In Ethiopia, there are strong cultural beliefs that deter open discussion on sexual and reproductive health, leading to misconceptions. Specifically, youths, are disadvantaged because of societal pressure, limited access to accurate sexual and reproductive health information, and services (36). The economic disparities also have a considerable impact on sexual and reproductive health knowledge and maternal health services utilization. Moreover, other factors such as education, residence, employment status, and media access are also indirectly related to economic status (37).

Our study indicated that education was a spatially statistically significant predictor of the spatial variation of the correct KOC. In areas where education was a significant factor, a 1% increase in the proportion of formally educated women corresponded to a 23%–36% increase in correct KOC. This finding is supported by a systematic review (38) and by studies conducted in Africa (4, 11), Ghana (13), Nigeria (30), Haiti (2), and the United States (6). This might be due to the impact of education on knowledge of human physiology and biology since the human reproductive system has been included as part of the human biology subject curriculum in Ethiopia. However, regional inequalities in educational achievement, educational opportunities (31), and educational quality differences (39) may be responsible for the regional variation of education across regions of Ethiopia.

Household media exposure was another spatially significant explanatory variable. It had a spatially and statistically significant positive effect on the prevalence of correct KOC among reproductive-age women. However, its statistical significance varies across different regions of Ethiopia. In significant areas, a 1% increase in the proportion of women with media exposure increases the prevalence of correct KOC by a range of 34%–57%. This finding is supported by a study conducted among African women (11) and Nigeria (30). It could be related to the significant influence of mass media exposure on maternal health awareness through the generation, acquisition, or dissemination of knowledge (40).

Our result also revealed that rich wealth status had a positive spatial effect on correct KOC among reproductive age women in significant parts of Ethiopia, in which, in these significant areas, a 1% increase in the proportion of women from households with a rich wealth quintile increased the prevalence of correct KOC among reproductive age women by a range of 13%–54%. This finding is supported by evidence from Africa (11). This might be due to the relation between economic status and educational opportunity and achievements (41–43), where better economic status may enhance educational opportunity and achievement positively; and media exposure (44, 45), which is a way of disseminating health-related information, including FP methods (43, 46, 47).

Another important and significant spatial explanatory variable was distance to health facilities. The proportion of women residing near health facilities showed both positive and negative spatial effects on the correct KOC, which ranges from −0.402 to 0.227. The negative effect was seen in Addis Ababa, SNNPR, Oromia, some parts of Gambela, Benishangul Gumuz, and Amhara, where a 1% increase in the proportion of women residing near health facilities decreased the prevalence of correct KOC by a range of 24.3%–40.2%. This is a surprising finding. However, another study conducted in Africa also indicated that residing near health facilities had no significant effect (11). On the other hand, in Dire Dawa, Harari, eastern Oromia, and eastern and southeastern Somali, it showed a significant positive effect as the proportion of women residing near health facilities increased by 1% and the prevalence of correct KOC increased by 15%–22.7%. This variation might be contributed to health extension program quality and implementations across the regions (34). Moreover, the negative effect seen might be due to the effect of customers’ large volume on the quality of primary health care, including counseling regarding the reproduction cycle (48).

Our study revealed that high community family planning message exposure is another spatial predictor for correct KOC. A spatially positive effect was seen in significant areas, in which a 1% increase in the proportion of women from clusters of high community FP messages in these areas can increase correct KOC by a range of 28%–39%. Which is supported by previous evidence from Cameroon (49) and Haiti (2). Surprisingly, in some parts of Benishangul and western Oromia, when the proportion of women from clusters of high community FP messages increases by 1%, correct knowledge of KOC decreases by a range of 2.2%–16%. This might be due to differences in health-related message dissemination and the quality of health program implementation.

Our study implied that considering spatial analyses to examine the regional disparity of KOC and the spatial factors influencing the geographical variation may be helpful. This approach can help in pinpointing areas with low rates of KOC and developing context- and area-based interventions.

### Strength and limitations of the study

This study is based on nationally representative data collected with a validated instrument. We employed a spatial analysis to identify hotspot and cold spot areas of correct KOC and identify spatial explanatory variables for this significant spatial variation in a specific geographic area. However, the GPS position used in our study is randomly displaced in order to maintain respondent confidentiality. As a result, urban clusters contain 0–2 km, rural clusters contain 0–5 km, and 1% of the rural clusters contain 0–10 km of positional errors. This may affect the local estimates and make it challenging to pinpoint the actual location of the cases.

## Conclusion

In this study, correct KOC among reproductive-aged women was found to be low. Our findings indicate an important need for strategies to improve KOC among reproductive-age women. Therefore, increasing KOC among women of reproductive age should become a priority to reduce an increasing fertility rate and prevent unintended pregnancy and abortion in the country. Significant spatial variation of correct KOC among reproductive-age women across regions of Ethiopia was indicated. Both individual and community-level factors showed a significant relationship with the correct KOC. Therefore, area-based interventional strategies are required in cold-spot areas to enhance adequate KOC. In addition, considering spatial explanatory variables in the implementation of these strategies rather than random provision of service would make regional health care delivery services more cost-effective. Lastly, our finding should encourage public health authorities to develop targeted reproductive education, which can be delivered effectively at schools, because it will help them understand their reproductive physiology.

## Data Availability

Publicly available datasets were analyzed in this study. This data can be found here: http://www.dhsprogram.com.
